# Methodology in core outcome set (COS) development: the impact of patient interviews and using a 5-point versus a 9-point Delphi rating scale on core outcome selection in a COS development study

**DOI:** 10.1186/s12874-020-01197-3

**Published:** 2021-01-07

**Authors:** Alexandria Remus, Valerie Smith, Francesca Wuytack

**Affiliations:** School of Nursing and Midwifery, Trinity College Dublin, Dublin, Ireland

**Keywords:** Core outcome set, Rating scales, Delphi methods, Consensus methods, Pelvic girdle pain, Patient interviews

## Abstract

**Background:**

As the development of core outcome sets (COS) increases, guidance for developing and reporting high-quality COS continues to evolve; however, a number of methodological uncertainties still remain. The objectives of this study were: (1) to explore the impact of including patient interviews in developing a COS, (2) to examine the impact of using a 5-point versus a 9-point rating scale during Delphi consensus methods on outcome selection and (3) to inform and contribute to COS development methodology by advancing the evidence base on COS development techniques.

**Methods:**

Semi-structured patient interviews and a nested randomised controlled parallel group trial as part of the Pelvic Girdle Pain Core Outcome Set project (PGP-COS). Patient interviews, as an adjunct to a systematic review of outcomes reported in previous studies, were undertaken to identify preliminary outcomes for including in a Delphi consensus survey. In the Delphi survey, participants were randomised (1:1) to a 5-point or 9-point rating scale for rating the importance of the list of preliminary outcomes.

**Results:**

Four of the eight patient interview derived outcomes were included in the preliminary COS, however, none of these outcomes were included in the final PGP-COS. The 5-point rating scale resulted in twice as many outcomes reaching consensus after the 3-round Delphi survey compared to the 9-point scale. Consensus on all five outcomes included in the final PGP-COS was achieved by participants allocated the 5-point rating scale, whereas consensus on four of these was achieved by those using the 9-point scale.

**Conclusions:**

Using patient interviews to identify preliminary outcomes as an adjunct to conducting a systematic review of outcomes measured in the literature did not appear to influence outcome selection in developing the COS in this study. The use of different rating scales in a Delphi survey, however, did appear to impact on outcome selection. The 5-point scale demonstrated greater congruency than the 9-point scale with the outcomes included in the final PGP-COS. Future research to substantiate our findings and to explore the impact of other rating scales on outcome selection during COS development, however, is warranted.

**Supplementary Information:**

The online version contains supplementary material available at 10.1186/s12874-020-01197-3.

## Background

Recently, there has been an increase in the development of core outcome sets (COS) to overcome the heterogeneity in outcome selection across clinical trials for a broad spectrum of health conditions [1]. A COS is a standardised set of outcomes which should be measured and reported, as a minimum, in all studies for a specific health area/condition [2]. The standardised set of outcomes allows for the results across trials to be combined or compared, reduces the potential for reporting bias and ensures that outcomes are meaningful, relevant and useable. Use of COS in trials and systematic reviews, can assist and strengthen the evidence base, resulting in improved quality of care worldwide. The development of a COS is a stepwise process that involves working with relevant stakeholders of a particular health condition/area to prioritise the core set from a larger list of outcomes which have been identified through earlier work [2]. Guidance for developing and reporting high-quality COS is evolving, however a number of methodological uncertainties still remain [2–6].

Involving patients/health service users from an early stage is recommended in COS development; however, the most appropriate way to facilitate inclusion remains largely unknown [2, 7]. Participation in Delphi surveys is the most popular method used for patient inclusion by COS developers, but mixed methods techniques are becoming increasingly popular [7, 8]. COS developers using mixed method techniques often conduct patient interviews as an adjunct to a systematic review of the literature to identify an initial list of potential outcomes for inclusion in a Delphi consensus survey [9]. This reflects current COS development guidance which recommends that the initial list of outcomes is identified from multiple sources including systematic reviews of published studies, reviews of qualitative work, examination of items collected in national audit data sets and interviews or focus groups with key stakeholders, such as patients [2]. In addition to helping identify potential outcomes for a COS, patient interviews may also assist research teams in understanding why particular outcomes are so important and also in understanding the language used by patients when referring to these outcomes in other phases of COS development [9]. However, conducting these patient interviews increases the workload and adds additional costs, resources and time for the COS development team in the absence of clear evidence of impact on final outcome selection; as such, research on this topic is recommended [2, 10].

After the preliminary list of outcomes has been identified, the Delphi technique is the most commonly used method for rating the importance of these outcomes for including in the COS [2]. The Delphi is an iterative survey method whereby relevant stakeholders are sent a series of questionnaires, known as ‘rounds’, and are asked to rate the importance of each identified outcome for inclusion in the COS on a scale of some description, usually using a rating scale. The Delphi technique is advantageous because it allows individuals to respond anonymously and can be circulated to a large number of diverse stakeholders without any geographical restrictions [11]. The COMET (Core Outcome Measures in Effectiveness Trials) Initiative provides guidance for using the Delphi technique to prioritise outcomes in developing a COS, but recognises also that there are a number of methodological uncertainties surrounding this method which need to be further explored [2, 4, 12]. For example, a variety of rating scales have been used in COS development. However, it remains unclear which rating scale is the most appropriate for use in the Delphi phase of a COS development study. Qualitative interviews reported mixed feedback from user experience of different rating scales [13] and only one study has compared the use of two different rating scales, a 3-point and a 9-point scale, for rating preliminary outcomes [14]. The authors of this study reported that the use of the 9-point rating scale resulted in almost twice as many outcomes being rated as important compared with the 3-point rating scale in the first Delphi round. Too many outcomes after each Delphi round is challenging because the goal of this process is to narrow down a larger list into a minimum set and a COS with too many outcomes may not be feasible or may not be ultimately adopted in research and clinical practice. For this reason, we embedded a randomised trial within our Pelvic Girdle Pain Core Outcome Set (PGP-COS) development project to compare the impact of a 5-point versus a 9-point rating scale on preliminary outcome selection and the final agreed COS [10, 15].

The objectives of this study were:

1. To determine if including patient interviews as an adjunct to systematic review for identifying the initial list of outcomes influences the final COS.
2. To evaluate the use of a 9-point versus a 5-point rating scale in the Delphi phase of a COS development study on the number of “important” ratings received for each outcome in each round of the Delphi and on the final COS, as well as their impact on attrition rates and their ease and clarity of use.
3. To inform and contribute to COS development methodology by advancing the evidence base on COS development techniques.

## Methods

### Material and methods

This study was embedded within the Pelvic Girdle Pain (PGP-COS) study and its protocol was published prospectively [10]. Ethical approval for the study was granted by the University Research Ethics Committee. The PGP-COS was developed by undertaking initial work to first identify potential outcomes through a systematic review of previous studies and semi-structured patient interviews, followed by inviting stakeholders to rate the importance of these outcomes for inclusion in the PGP-COS in a 3-round Delphi survey, and, finally, by agreeing on the final COS in a face-to-face consensus meeting with key stakeholders. In depth methodological details about the design and analysis of the PGP-COS project, including the systematic review, semi-structured interviews, the Delphi survey and the consensus meeting are available in the study protocol [10], the published systematic review [16] and in the PGP-COS main report (Remus et al: A Core outcome set for research and clinical practice in women with pelvic girdle pain: PGP-COS, Under review). For flow and clarity of the summary details of the initial work leading to the Delphi and the embedded randomised trial are described below.

### Steering committee

An International Steering Committee with members from five countries, including researchers, clinicians, and methodologists worked on the development of this COS. The day-to-day conduction of the study was performed by a project team of three people (AR, FW, VS) working at the same institution (Trinity College Dublin, Ireland) who designed and addressed key aspects of the study. The other members of the Committee were involved in conducting interviews, participated in meetings to discuss the progress and monitor the conduct of the study and provided consultation regarding critical decisions.

### Interviews

Interviews of 15 women with experience of PGP, either presently or previously, in three countries; Ireland (*n* = 5), Sweden (*n* = 5) and Mexico (*n* = 5), were undertaken to seek patient’s views on their treatment needs and PGP outcomes that were important to them. Participants were recruited via physiotherapy and chiropractic clinics and provided written informed consent for taking part in the interviews. The phase of the study was descriptively qualitative (Remus et al: A Core outcome set for research and clinical practice in women with pelvic girdle pain: PGP-COS, Under review).

### Delphi study

**Fig. 1:**
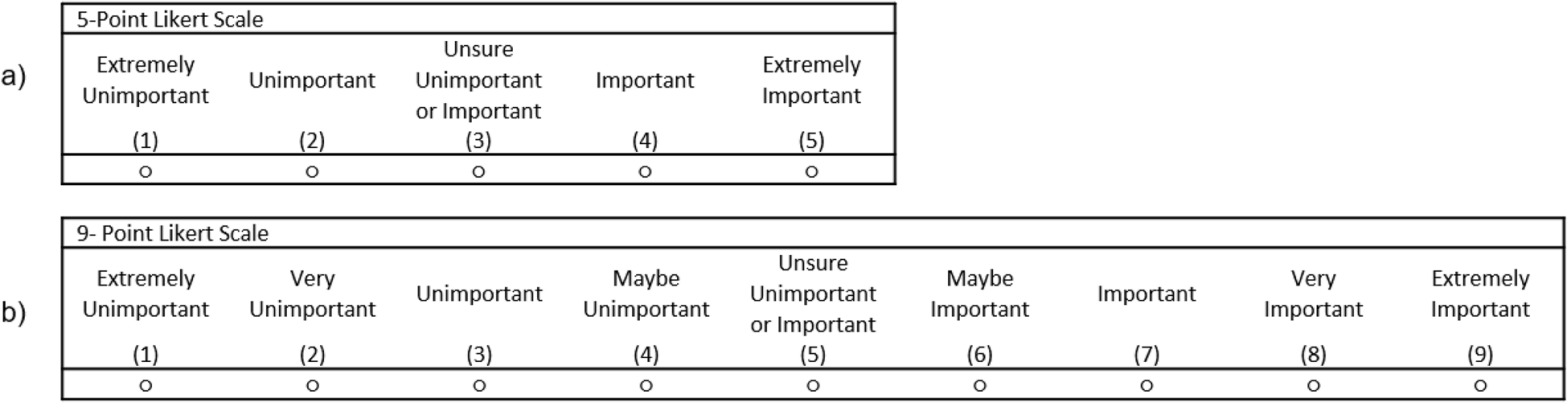
Delphi questionnaire rating scales

The systematic review searched for and extracted the outcomes reported in all previous intervention studies on PGP and lumbar-pelvic pain. One-hundred and seven studies were included in the review, yielding 45 distinct outcomes [16]. These outcomes were then grouped into core domains using the OMERACT filter 2.0 framework [17]. The systematic review and patient interviews collectively generated a list of 53 preliminary outcomes which were entered into a bespoke Delphi questionnaire created in Google forms [18]. Two versions of this questionnaire were created: one with a 5-point rating scale and one with a 9-point rating scale (Fig. 1). Five stakeholder groups including PGP patients, clinicians, researchers, dual role researcher and clinician, and policy makers/service providers were invited to participate via mass invitational emails to patient and professional organisations and through social media (Facebook and Twitter) using snowball sampling methods.

### Choosing the rating scales

In meeting objective 2 we first had to choose our comparator rating scales, noting that the type of rating scale used can present different data collection and analytical challenges and may give rise to concerns for data quality especially if the scale has comparable validity and reliability issues [19]. For example, a scale offering too few options, such as ‘agree’ and ‘disagree’ will not capture ‘neutral’ or ‘unsure’ attitudes. Adding further options, including moderate options at the positive and negative ends of the scale (e.g. ‘somewhat agree’) will allow for greater differentiation in user judgement resulting, potentially, in more accurate representation of attitudes. Concurrently, if too many options are offered, the clarity of the scale might become compromised, as each additional point is one more point that the user must interpret [19].

Earlier empirical research on rating scales has demonstrated various results with respect to the reliability and validity of scales of different lengths. Alwin and Krosnick identified a 3-point scale as having the lowest reliability, 2, 4, 5, and 7-point scales as having equivalent reliability, and 9-point scales as having maximum reliability [20]. Contrastingly, Scherpenzeel found the highest reliability for 5-point scales and lower reliability for 10-point or longer scales [21]. Other studies also note increasing reliability from 2 to 3 to 5-point scales but equivalent, or minimal increases, thereafter for 7, 9, and 11-point scales [22, 23]. In a study that asked participants to rate various objects by marking points on lines with no discrete category divisions and to indicate their range around each judgement, the estimated number of scale points naturally employed was 5 [24], although when more scale points were offered (up to 19), the more points people used, up to about 9 [25].

Although this evidence is informative and supportive for the scales chosen in the current study, ultimately the 9-point scale was chosen because it is recommended by the Grading of Recommendations Assessment, Development and Evaluation (GRADE) working group to assess the importance of evidence and is the scale used in DelphiManager, a web-based system designed by the COMET Initiative for facilitating and managing Delphi surveys [2, 26, 27]. The 5-point rating scale was arbitrarily chosen as the comparator scale because it had been used previously in COS development Delphi studies [28–30], and because 5-point rating scales are relatively common across research studies that include a rating scale.

### Intervention

All Delphi participants were randomly allocated to one of the two survey versions when they clicked the link to participate in round 1 of the Delphi study. Random survey allocation was achieved using a random redirect tool published online (www.allocate.monster). When a participant accessed the link created by the tool, the background code selected one of the two versions of the questionnaires at random and redirected the participant to that version. The randomisation method was simple randomisation [31]. Participants used the same rating scale they were initially allocated for all three Delphi rounds.

The Delphi surveys were typical of those previously used in COS development with participants being asked to rate the importance of each outcome for inclusion in the PGP-COS in each round using the scale they were allocated to. After each round all participants were emailed a copy of their responses for reference in subsequent rounds. In rounds 2 and 3, participants were provided with the proportion of participants in each stakeholder group who rated each outcome as “important” [4+ (5-point survey) and 7+ (9-point survey)]. In round 1, participants were also given the opportunity to suggest up to a maximum of three additional outcomes using a free-text response. The additional outcomes identified from both survey versions were combined so that all participants using either survey version had the opportunity rate these outcomes in round 2, in addition to rerating all outcomes included in round 1. After round 2, only outcomes that reached a priori consensus, that was ≥70% participants scoring the outcome as “important” [4+ (5-point scale) or 7+ (9-point scale)] for 3/5 stakeholder groups, inclusive of the patient representative group, were included in the round 3 Delphi surveys. During the Delphi phase, each survey was treated independently. Following round 3, all outcomes that reached a priori consensus on either of the survey versions were included in a preliminary PGP-COS. These two preliminary PGP-COSs were then combined as one list of outcomes and presented at the face-to-face consensus meeting, where key stakeholders (i.e. at least one representative from each of the Delphi survey groups, and 11 stakeholders in total) voted on the outcomes for inclusion in the final PGP-COS. Additionally, at the end of round 3, questions on the ease of use, ease of understanding, and the clarity of the scale were posed to participants, with an opportunity to provide any additional comments about the survey using free-text.

### Sample size

The embedded trial was based on an opportunistic sample of all 205 participants involved in the Delphi phase of the main PGP-COS development project. Therefore, no sample size calculations were performed as statistical analysis was intended to be exploratory and formative.

### Data analysis

#### Analysis 1: influence of patient interview-derived outcomes

To investigate objective 1, we analysed the following: the number of new outcomes identified in patient interviews, how the interview-derived outcomes were rated in each Delphi round, the number of interview-derived outcomes included in the final COS and the extent to which additional outcomes provided by patients in round 1 of the Delphi survey overlapped with the interview-derived outcomes. All descriptive statistical analyses were performed using Excel (Microsoft Excel 2016).

#### Analysis 2: influence of Delphi rating scale on final COS

We used descriptive statistics (counts and %) for demographic and survey response data. To investigate objective 2 we analysed the following: the proportion of outcomes reaching a priori consensus in each survey in each round, the proportion of outcomes included in the preliminary COS for each survey, differences between the scales whether the outcomes included in the final COS had reached consensus in each survey and attrition rate for each scale. Z-scores were calculated to test the differences in proportion of outcomes reaching a priori consensus and overall attrition between the surveys with alpha set to 0.05 using the formula below [32]:

$$ Z=\frac{\left(p1-p2\right)-0}{\sqrt{p\left(1-p\right)\left(\frac{1}{n1}+\frac{1}{n2}\right)}} $$

Whereby p1 = proportion from 5-point survey; p2 = proportion from 9-point survey; n1 = number of possible outcomes from 5-point survey; n2 = number of possible outcomes from 9-point survey; and p = pooled proportion. All statistical analyses were performed using Excel (Microsoft Excel 2016) [33]. Scale “ease of use” and “clarity” responses plus any additional comments were analysed using quantitative content analysis.

## Results

**Fig. 2:**
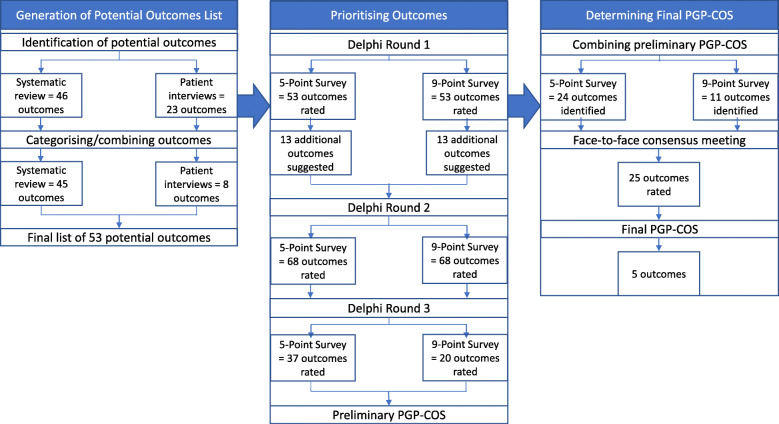
An overview of the PGP-COS study and results

Figure 2 presents an overview of the PGP-COS phases and the summary results of the embedded studies.

### Analysis 1: influence of patient interview-derived outcomes

The fifteen patient interviews identified 23 outcomes for inclusion in the round 1 Delphi questionnaire. Fifteen of these outcomes overlapped with outcomes identified from the systematic review, and eight were new outcomes [16]. The patient interview-derived new outcomes were pain character/type, need for a mobility aid, perceived body imbalance, sexual functioning, family life impact, social life impact, emotional symptoms and frustration. Table 1 shows how stakeholders rated the interview-derived outcomes in all three rounds of the Delphi study. Four of the outcomes (50%) were included in the preliminary PGP-COS. None of the patient interview-derived outcomes were included in the final PGP-COS (Remus et al: A Core outcome set for research and clinical practice in women with pelvic girdle pain: PGP-COS, Under review). It should be noted, however, that due to travel complications only one participant representing the patient group was able to attend the face-to-face consensus meeting. However, five participants who identified primarily with a different stakeholder group also identified themselves as patients; i.e. patient/clinician (*n* = 1); patient/researcher (*n* = 2); patient/researcher/clinician (*n* = 1); patient/clinician/service provider (*n* = 1)).

**Table 1:** Stakeholder ratings of interview derived outcomes

		Round 1		Round 2		Round 3	
		*Survey*		*Survey*		*Survey*	
Outcome	Stakeholder Group	5 PT (%)^a^	9PT (%)^a^	5 PT (%)^a^	9PT (%)^a^	5 PT (%)^a^	9PT (%)^a^
Pain character/type	*Clinician*	77	67	81	83	82	72
	*Clinician/Researcher*	61	40	68	43	42	29
	*Patient*	90	73	86	83	70	63
	*Researcher*	60	54	50	36	30	27
	*Service Provider/Policy Maker*	100	67	100	80	67	80
Need for a Mobility Aid	*Clinician*	81	73	84	72	75	–
	*Clinician/Researcher*	70	53	74	43	58	–
	*Patient*	85	73	79	75	60	–
	*Researcher*	80	46	60	64	20	–
	*Service Provider/Policy Maker*	100	67	67	60	100	–
Perceived Body Imbalance	*Clinician*	47	56	26	34	–	–
	*Clinician/Researcher*	48	33	26	29	–	–
	*Patient*	70	68	57	58	–	–
	*Researcher*	40	15	0	27	–	–
	*Service Provider/Policy Maker*	100	17	33	40	–	–
Sexual Functioning^b^	*Clinician*	86	79	87	79	75	–
	*Clinician/Researcher*	70	67	84	79	68	–
	*Patient*	80	64	79	42	70	–
	*Researcher*	80	69	70	73	70	–
	*Service Provider/Policy Maker*	100	83	67	60	100	–
Family Life Impact^b^	*Clinician*	93	79	97	93	93	84
	*Clinician/Researcher*	87	73	79	71	95	50
	*Patient*	90	91	100	83	100	63
	*Researcher*	90	85	100	73	100	64
	*Service Provider/Policy Maker*	100	83	100	60	100	80
Social Life Impact^b^	*Clinician*	91	81	87	83	71	80
	*Clinician/Researcher*	87	67	84	64	84	50
	*Patient*	85	86	86	75	80	50
	*Researcher*	80	85	90	82	90	55
	*Service Provider/Policy Maker*	100	83	67	80	100	100
Emotional Symptoms^b^	*Clinician*	74	81	74	90	75	–
	*Clinician/Researcher*	83	67	79	64	79	–
	*Patient*	80	86	79	83	80	–
	*Researcher*	90	77	70	55	70	–
	*Service Provider/Policy Maker*	60	83	67	40	33	–
Frustration	*Clinician*	72	67	35	55	–	–
	*Clinician/Researcher*	43	40	32	7	–	–
	*Patient*	80	82	71	67	–	–
	*Researcher*	40	23	40	27	–	–
	*Service Provider/Policy Maker*	60	50	67	60	–	–

^a^% of each stakeholder groups that rated the outcome as “important” on each scale (5 point and 9 point); (−) Indicates that the outcome did not go through to the corresponding Delphi survey round and subsequently was not rated by stakeholder groups; ^b^ Denotes outcomes that were included in the preliminary COS after round 3 of the Delphi

During round 1 of the Delphi study, patients suggested 16 additional outcomes using the free-text option, of which, 11 were considered actual outcomes by the PGP-COS study steering committee representatives (AR, FW) and four (36%) as new outcomes which were subsequently included in round 2 of the Delphi study. Six of the 11 outcomes (55%) overlapped with outcomes identified in the systematic review and 1 outcome (9%) overlapped with a patient interview-derived outcome. None of the additional outcomes suggested by patients were included in the final PGP-COS. Table 2 presents the suggested additional outcomes and decisions pertaining to these.

**Table 2:** Additional outcomes

Patient Suggested Additional Outcomes	Outcome Name	Steering Committee Group Decision	Comparison to Other Outcomes
“Alternative therapy - impact and access to - some medications cannot be used...”	n/a	Not an outcome but included in definition of healthcare utilisation	–
“Likelihood of planning subsequent pregnancies, due to risk of reoccurrence. ”	Likelihood of planning subsequent pregnancies	Include	New outcome
“Onset of PGP: pregnancy week or postpartum”	n/a	Not an outcome	–
“Maternal comorbidities such as Ehlers-Danlos or diabetic. Family related PGP”	n/a	Not an outcome - risk factor	–
“In ability to bond with child, relationship damage”	Family life Impact	Already included	Interview-derived outcome
“Information on Lactation for how long, problems, in which positions.”	Function	Already included	Systematic review outcome
“Menstruation when returned after birth and what about PGP in connection with menstruation”	Symptoms during menstruation	Include	New outcome
“Postnatal experiences of PGP”	n/a	Not an outcome	–
“Length of time to become pain free again”	Full symptom recovery	Already included	Systematic review outcome
“Mattress - what quality, need of overlay that reduce pressure on the pelvic joints, need of mattress filled with water. Need of cushions like the ROHO or other kind of cushions that relieve pressure on the pelvic girdle when sitting.”	Needs for additional supports	Include	New outcome
“Need for help with taking care of the baby, need of daycare for older siblings”	Dependence on others	Already included	Systematic review outcome
“Need for help with the daily work in the house like making dinner, cleaning, washing clothes and so on”	Activity Daily Life	Already included	Systematic review outcome
“Perseverance in standing”	Function	Covered Already included	Systematic review outcome
“Accessibility of treatment”	n/a	Not an outcome	–
“How your own hormones affects the ability to do things, and how it affects the pain. How a hormone spiral optionally may influence on the ability to do things”	n/a	Not an outcome	–
“Fitness levels prior to pain onset”	Exercise/activity levels	Already included	Systematic review outcome
“How long the PGP pain has lasted”	Pain duration	Include	New outcome

### Analysis 2: influence of Delphi rating scale

Participant demographics for all three rounds of the Delphi study can be viewed in Table 3. An overview of outcome inclusion and exclusion is detailed in Fig. 2. Comparison of outcomes reaching a priori consensus between the two survey versions for each Delphi round are detailed in Table 4.

**Table 3:** Delphi Study Participant Demographics

	Round 1		Round 2		Round 3	
	5-point *n = 101*	9-point *n = 104*	5-point *n = 76*	9-point *n = 71*	5-point *n = 69*	9-point *n = 63*
**Stakeholder Group** ***n (%)***						
Clinician	43 (43%)	48 (46%)	30 (39%)	29 (41%)	27 (39%)	25 (40%)
Clinician Researcher	23 (23%)	15 (14%)	19 (25%)	14 (20%)	19 (28%)	14 (22%)
Patient	20 (20%)	22 (21%)	14 (18%)	12 (17%)	10 (14%)	8 (13%)
Researcher	10 (10%)	13 (13%)	10 (13%)	11 (15%)	10 (14%)	11 (17%)
Service Provider/Policy Maker	5 (5%)	6 (6%)	3 (4%)	5 (7%)	3 (4%)	5 (8%)
**Gender** ***n (%)***						
Female	73 (72%)	86 (83%)	57 (75%)	57 (80%)	51 (74%)	50 (79%)
Male	27 (27%)	18 (17%)	19 (25%)	14 (25%)	18 (26%)	13 (21%)
Prefer not to say	1 (0%)	0 (0%)	0 (0%)	0 (0%)	0 (0%)	0 (0%)
**Age** ***n (%)***						
18–24	2 (2%)	1 (1%)	1 (1%)	0 (0%)	1 (1%)	0 (0%)
25–34	19 (19%)	22 (21%)	16 (21%)	12 (17%)	13 (19%)	9 (14%)
35–44	34 (34%)	40 (38%)	23 (30%)	28 39%)	20 (29%)	24 (38%)
45–54	24 (24%)	20 (19%)	19 (25%)	16 (23%)	18 (26%)	15 (24%)
55–64	18 (18%	15 (14%)	15 (20%)	11 (15%)	15 (22%)	11 (17%)
65+	4 (4%)	6 (6%)	2 (3%)	4 (6%)	2 (3%)	4 (6%)
**Country** ***n (%)***						
Argentina	1 (1%)	0 (0%)	0 (0%)	0 (0%)	0 (0%)	0 (0%)
Australia	4 (4%)	4 (4%)	3 (4%)	3 (4%)	3 (4%)	3 (5%)
Austria	1 (1%)	0 (0%)	1 (1%)	0 (0%)	1 (1%)	0 (0%)
Belgium	3 (3%)	1 (1%)	3 (4%)	1 (1%)	3 (4%)	1 (2%)
Brazil	1 (1%)	0 (0%)	1 (1%)	0 (0%)	1 (1%)	0 (0%)
Canada	6 (6%)	11 (11%)	4 (5%)	9 (13%)	4 (6%)	9 (14%)
Colombia	1 (1%)	0 (0%)	1 (1%)	0 (0%)	1 (1%)	0 (0%)
Cook Islands	0 (0%)	1 (1%)	0 (0%)	0 (0%)	0 (0%)	0 (0%)
Croatia	1 (1%)	0 (0%)	0 (0%)	0 (0%)	0 (0%)	0 (0%)
Denmark	1 (1%)	2 (2%)	1 (1%)	2 (3%)	1 (1%)	2 (3%)
Egypt	0 (0%)	1 (1%)	0 (0%)	0 (0%)	0 (0%)	0 (0%)
Finland	1 (1%)	0 (0%)	1 (1%)	0 (0%)	1 (1%)	0 (0%)
Germany	1 (1%)	0 (0%)	1 (1%)	0 (0%)	1 (1%)	0 (0%)
Iceland	1 (1%)	0 (0%)	0 (0%)	0 (0%)	0 (0%)	0 (0%)
Iran	0 (0%)	2 (2%)	0 (0%)	2 (3%)	0 (0%)	2 (3%)
Ireland	26 (26%)	19 (18%)	18 (24%)	13 (18%)	14 (20%)	10 (16%)
Israel	1 (1%)	0 (0%)	1 (1%)	0 (0%)	1 (1%)	0 (0%)
Malaysia	4 (4%)	0 (0%)	2 (3%)	0 (0%)	2 (3%)	0 (0%)
Mexico	0 (0%)	3 (3%)	0 (0%)	3 (4%)	0 (0%)	2 (3%)
Nepal	2 (2%)	0 (0%)	2 (3%)	0 (0%)	2 (3%)	0 (0%)
Netherlands	3 (3%)	1 (1%)	3 (4%)	1 (1%)	3 (4%)	1 (2%)
New Zealand	1 (1%)	2 (2%)	1 (1%)	1 (1%)	1 (1%)	1 (2%)
Norway	7 (7%)	7 (7%)	7 (9%)	4 (6%)	7 (10%)	4 (6%)
Philippines	0 (0%)	1 (1%)	0 (0%)	0 (0%)	0 (0%)	0 (0%)
Poland	0 (0%)	1 (1%)	0 (0%)	1 (1%)	0 (0%)	1 (2%)
Portugal	1 (1%)	2 (2%)	1 (1%)	1 (1%)	1 (1%)	1 (2%)
South Africa	0 (0%)	1 (1%)	0 (0%)	1 (1%)	0 (0%)	1 (2%)
Sweden	11 (11%)	15 (14%)	9 (12%)	9 (13%)	9 (13%)	9 (14%)
Switzerland	1 (1%)	3 (3%)	1 (1%)	3 (4%)	0 (0%)	3 (5%)
UK	11 (11%)	17 (16%)	8 (11%)	9 (13%)	7 (10%)	5 (8%)
USA	11 (11%)	9 (9%)	7 (9%)	7 (10%)	6 (9%)	7 (11%)
Zimbabwe	0 (0%)	1 (1%)	0 (0%)	1 (1%)	0 (0%)	1 (2%)

**Table 4:** Selection of outcomes in Delphi study

	**Round 1**		**Round 2**		**Round 3**	
**Outcome**	**5PT**	**9PT**	**5PT**	**9PT**	**5PT**	**9PT**
Pain behaviour	In	Out	Out	Out	–	–
Pain character/type	In	Out	In	In	Out	Out
Pain frequency	In	In	In	In	In	In
Pain intensity/severity	In	In	In	In	In	In
Pain location	In	In	In	In	In	In
Full pain recovery	In	In	In	In	In	Out
Function/disability/activity limitation	In	In	In	In	In	In
Physical activity levels/exercise limitations	In	In	In	In	In	In
Need for mobility aid	In	Out	In	Out	Out	–
Perceived body imbalance	Out	Out	Out	Out	–	–
Sexual functioning	In	Out	In	Out	In	–
Health related quality of life	In	In	In	In	In	In
Health status	In	In	In	In	In	Out
Family life impact	In	In	In	In	In	Out
Social life impact	In	In	In	In	In	Out
Patient satisfaction with life	In	In	In	In	In	Out
Patient satisfaction with treatment	In	In	In	In	In	In
Patient expectations of treatment	In	In	Out	In	–	In
Anxiety	In	In	In	In	In	Out
Confidence	In	In	Out	Out	–	–
Depression	In	In	In	Out	In	–
Dependence on others	In	In	Out	Out	–	–
Emotional symptoms	In	In	In	Out	In	–
Fear avoidance	In	In	In	In	In	Out
Frustration	Out	Out	Out	Out	–	–
Pain catastrophizing	In	In	In	Out	In	–
Self-efficacy	In	In	In	Out	In	–
Well-being	In	In	Out	Out	–	–
Fatigue	In	Out	Out	Out	–	–
Sleep function	In	In	In	In	In	Out
Work ability	In	In	In	In	In	In
Work performance	In	In	In	Out	Out	–
Analgesia use	In	Out	Out	Out	–	–
Cost	In	In	Out	Out	–	–
Healthcare utilisation	In	In	In	Out	Out	–
Anthropomorphic outcomes	Out	Out	Out	Out	–	–
Body flexibility	Out	Out	Out	Out	–	–
Functional mobility	In	Out	Out	Out	–	–
Gait endurance	In	Out	In	Out	In	–
Gait speed	Out	Out	Out	Out	–	–
New-born outcomes	Out	Out	Out	Out	–	–
Outcomes from pain provocation/location tests	In	Out	Out	Out	–	–
Posture	In	Out	Out	Out	–	–
Pubis symphysis mobility	Out	Out	Out	Out	–	–
Maternal pregnancy outcomes	Out	Out	Out	Out	–	–
Muscle endurance	Out	Out	Out	Out	–	–
Muscle strength	In	Out	Out	Out	–	–
Recovery of symptoms	In	In	In	In	In	In
Step length	Out	Out	Out	Out	–	–
Surgical outcomes	Out	Out	Out	Out	–	–
Urinary Incontinence	In	In	In	Out	In	–
Maternal adverse outcomes/undesirable effects	In	Out	In	Out	Out	–
Unborn/born adverse outcomes/ undesirable effects	Out	Out	Out	Out	–	–
Breathing function	–	–	Out	Out	–	–
Clinical findings on motion palpation/joint play of pelvic girdle joints	–	–	Out	Out	–	–
Coping strategies/coping styles	–	–	In	Out	Out	–
Global perceived improvement/global rate of change	–	–	In	Out	Out	–
Goal attainment	–	–	In	Out	Out	–
	**Round 1**		**Round 2**		**Round 3**	
**Outcome**	**5PT**	**9PT**	**5PT**	**9PT**	**5PT**	**9PT**
Likelihood of planning subsequent pregnancies	–	–	Out	Out	–	–
Motor control/movement strategies/ movement patterns	–	–	In	Out	In	–
Muscle tightness	–	–	Out	Out	–	–
Need for additional supports	–	–	Out	Out	–	–
Outcomes from functional tests	–	–	In	Out	–	–
Pain duration/pain pattern	–	–	In	In	In	In
Patients beliefs about pain/meaning of complaints to patient	–	–	In	Out	Out	–
Patient understanding/knowledge of PGP	–	–	In	Out	Out	–
Postural observation	–	–	Out	Out	–	–
Symptoms during menstruation	–	–	Out	Out	–	–
Possible outcomes in each round *n*	53	53	68	68	37	20
Total “In” each round *n*	41	29	37	20	24	11
% “In” each round	77%	55%	54%	29%	65%	55%
Z-score (outcomes “in”)	2.46		2.95		0.73	

Selection of outcomes on the 5-point scale survey version and the 9-point scale survey version for all Delphi rounds based on the criterion of ≥70% participants scoring the outcome as “important” [4+ (5-point scale) or 7+ (9-point scale)] for 3 of the 5 stakeholder groups, inclusive of the patient representative group

- Denotes the outcome was not rated in that particular Delphi round

There was a significant difference in the proportion of outcomes reaching a priori consensus between the two scale versions in round 1 (Z = 2.46, *p = 0.01*) and round 2 (Z = 2.95, *p* = 0.00) of the Delphi study. After round 1, consensus was reached on 41 outcomes (77%) on the 5-point survey compared with 29 outcomes (55%) on the 9-point survey. After round 2, consensus was reached on 37 of the 68 round 2 outcomes (54%) on the 5-point survey compared with 20 outcomes (29%) on the 9-point survey. After Delphi round 3, there was a significant difference in the proportion of outcomes included in the preliminary PGP-COS between the two survey versions (Z = 2.55, *p* = 0.01). The resulting 5-point preliminary PGP-COS included 24 outcomes (35% of all round 3 outcomes); contrastingly the 9-point scale provided 11 outcomes (16%) for the preliminary PGP-COS. Ten of the 11 outcomes (91%) on the 9-point preliminary PGP-COS overlapped with outcomes from the 5-point preliminary PGP-COS. All five outcomes of the final PGP-COS were included in the 5-point preliminary PGP-COS, whereas, four outcomes from the final PGP-COS were included in the 9-point preliminary PGP-COS.

There was no difference in overall attrition between the two surveys (Z = 1.15, *p* = 0.25). Attrition rate for respondents using the 5-point scale was 25% between round 1 and round 2, 9% between round 2 and round 3 and 32% overall. Attrition rate for respondents using the 9-point scale was 32% between round 1 and round 2, 11% between round 2 and round 3 and 39%. Table 5 details attrition rates for per stakeholder group.

**Table 5:** details attrition rates for per stakeholder group

	***5-Point Survey***			***9-Point Survey***		
Stakeholder Group	R1-R2	R2-R3	Overall	R1-R2	R2-R3	Overall
Clinician	30%	10%	37%	40%	14%	48%
Clinician/Researcher	17%	0%	17%	7%	0%	7%
Patient	30%	29%	50%	45%	33%	64%
Researcher	0%	0%	0%	15%	0%	15%
Service Provider/Policy Makers	40%	0%	40%	17%	0%	17%
**Overall**	**25%**	**9%**	**32%**	**32%**	**11%**	**39%**

*R* Round

Delphi respondents who completed all 3 rounds only were invited to provide feedback on the ease of use, understanding and clarity of the rating scales by asking participants in round 3 if their allocated scale was easy to use and clear to understand. Feedback on the ease of use of both the 5-point scale and 9-point scale revealed that 64% (45/70) of people said the 5-point scale was easy/very easy to use while this was 51% (33/64) for the 9-point scale. However, for the 9-point scale people had to scroll across to see the all options which was commented on by 10 (16%) people as not being practical. For the 5-point scale, four people (6%) suggested more options would be useful while one person would have preferred a 3-point scale. For the 9-point scale, eight people (13%) thought there were too many options. Feedback on the clarity of both scales revelated that 87% (61/70) of respondents who provided feedback (as this was a non-obligatory question) found the 5-point scale very clear/clear to understand whereas this was 73% (47/64) for the 9-point scale.

## Discussion

This is the first embedded methodological study to examine the impact of patient interview-derived outcomes and to examine the comparison between a 5-point and 9-point rating scale on the development of a COS. Our embedded study found that none of the outcomes derived from the interviews only were included in the final PGP-COS. The rating scale used in the Delphi consensus process did influence the proportion of outcomes rated as “important” in the Delphi rounds and could potentially impact on a final COS whereby more or less outcomes would be available in the final COS consensus meeting.

### Patient interview-derived outcomes

It is plausible that conducting patient interviews to identify the initial list of outcomes for inclusion in the Delphi phase of COS development may not be as important as one might have thought, and especially, if resources and time are limited. At the face-to-face consensus meeting, it was discussed that three of the interview-derived outcomes were not deemed “absolutely critical” to be measured and reported in all trials as unique outcomes, but could be captured by two outcome measures that were included in the final PGP-COS. For example, ‘sexual functioning’ was considered important but participants agreed that this was covered in the outcome ‘functioning/disability/activity limitations’ of the final PGP-COS. This is important to consider when identifying the most appropriate instrument to measure these outcomes. It is essential that the instrument used measures all aspects of the outcomes relevant to patients. While patient interview derived outcome data were not included in the final COS in this study, they could still be valuable in providing insight into why outcomes are important to inform the consensus meeting. Overall, this highlights the importance of patient participation throughout the Delphi study and in face-to-face consensus meetings in the development of COS on “what” to measure as well as in later stages such as the development of “how” to measure the COS and suggests that it may not be necessary to conduct patient interviews to identify the initial list of potential outcomes.

Additionally, we identified that only one patient interview-derived outcome overlapped with the additional outcomes suggested by patients during round 1 of the Delphi study. However, due to our study design, the true extent of overlap between these outcomes remains unknown, as the initial list of potential outcomes was an amalgamation of the outcomes identified from the systematic review and interviews. We cannot ascertain if the other interview outcomes would have been suggested by patients if they were not included in the initial list that was presented during round 1 of the Delphi. Recommended Delphi methodology includes providing participants with the opportunity to suggest additional outcomes during the first survey round and asking participants to re-rate the initial list of outcomes in the subsequent round. A potential method for future studies to examine the full extent of overlap between these two outcomes would be to exclude any interview-derived outcomes from initial voting in the first round and introduce them in the second round of voting. This may offer more understanding on the extent of overlap between patient interview-derived outcomes and patient suggested additional outcomes in Delphi studies. It is plausible that patient interviews at the initial outcome identification stage are redundant if patients are included in the Delphi survey and in the consensus meeting.

### Impact of survey scales

In our study, the 5-point scale resulted in twice as many outcomes in the preliminary PGP-COS after three rounds compared with the 9-point scale. This could potentially be a downside to using a 5-point scale considering that the intention in using the series of survey rounds is reductionist based on outcomes being viewed as ‘critically important’ to the COS. It could also indicate that use of this scale in this PGP-COS had less discriminatory power for the outcomes offered for rating than the 9-point scale. This result, interestingly, is in direct contrast to that of De Meyer and colleagues who found twice as many outcomes selected as “critical” on the 9-point scale compared to the 3-point scale [14]. Although we cannot directly compare our results as scale differences were only explored in the first round of de Meyer’s Delphi and our consensus definitions differed, it is plausible that the sizeable difference in rating options between a 3-point and 9-point compared to a 5-point and 9-point may explain the conflicting results between our studies. In spite of the contrasting results, it is evident that the rating scale utilised in Delphi studies does impact on the outcomes made available to the final COS consensus meeting, and supports previous concerns raised for best practice when creating and using scales in survey research [19]. In particular, Bekstead identified that using a scale with too few response options may not allow a respondent to make full use of his or her capacity to discriminate while a scale with too many options may exceed the respondent’s capacity to discriminate, contributing to measurement error [34]. Collectively, the greater proportion of outcomes rated as important using the 5-point scale and the exclusion of one outcome from the final PGP-COS from the 9-point preliminary PGP-COS suggests that it may be harder to discriminate outcome importance rating when there are limited options to choose from and that too many options may result in measurement error. This is also supported by the free text responses provided by participants after our Delphi study which indicated that both scales were generally perceived as easy to use, although responses concerning the number of rating options were mixed on the 5-point scale but were generally reported as too many options on the 9-point scale. Additionally, we included combined fully labelled, numeric rating scales in our Delphi questionnaires, as fully labelled scales have been shown to produce more reliable and valid data [34]. In our surveys, our middle rating included the label of “unsure unimportant or important” for option 3 on the 5-point survey and 5 on the 9-point scale (Fig. 1). Participants sometimes expressed a lack of understanding of the middle rating option on both the 5-point and 9-point scales. As there is currently no established “gold standard” labelling system for rating scales, it is possible that the labels provided for the middle rating may have impacted clarity and understanding of the scale. It is also possible that a lack of understanding of the middle option may have influenced participants to select ratings on either end of the middle rating, thus inflating the number of outcomes rated as “important,” particularly on the 5-point scale. This may in turn, have impacted our results and should be considered when selecting a rating scale to use when prioritising outcomes. Overall, our results indicate that rating scales may impact the final COS because fewer, or more, outcomes can be made available to the consensus meeting following the Delphi process. Further research to explore the impact of the various commonly used rating scales, such as a 7-point scale, on the final COS is warranted.

In addition, our results highlight user experience concerns that research teams should consider when incorporating rating scales in COS protocols. For example, a number of our 9-point Delphi survey participants expressed that it was frustrating that they were required to scroll across to see all possible responses on the scale. This highlights the importance of user experience considerations when selecting a rating scale and also the platform in which a research group intends to use to disseminate their Delphi questionnaire, especially when a group intends to utilise e-Delphi surveys that are completed on mobile phones or computers with varying screen sizes. These considerations can be addressed with pilot testing of the surveys amongst end users before sending out the finalised e-Delphi surveys for data collection. It is important to note, however, that the comments regarding participant experience are only reflective of those who completed all three rounds of the Delphi. Additional insights could be derived from those who dropped out in earlier rounds; however, these were not collected. Future work should consider user experience during all rounds of a Delphi study. Our results also indicate the importance of designing a Delphi questionnaire that is user friendly with a goal of maintaining group involvement to completion if responses from this group is of importance. Although there was no difference in retention rates between rating scales, the patient-representative group had the highest rate of drop out on both surveys whilst researchers and clinician/researcher group representatives had the lowest attrition rates on both surveys. It is possible that researchers and clinician/researcher groups may have a stronger understanding of the implications of COS development and/or research methodology compared to other stakeholder groups, as explained by their high retention. These results combined with those from our patient interview analysis suggests that input from patient-group representatives should be considered in the initial questionnaire and provides the opportunity to include patients at an early stage in COS development. Overall, it is evident that COS development teams should also consider user experience when selecting an appropriate scale that best suits the target populations in COS methodology development.

### Limitations

This study had several limitations. This study was embedded within one COS development project only. The inclusion of patient interview-derived outcomes in the initial list used in the first Delphi round did not allow us to evaluate the full extent of overlap between the interview-derived outcomes and the additional outcomes suggested by patients. Only two rating scales were used to study the influence of scale selection on outcomes made available to the final COS. We compared a 5-point and 9-point scale only, both of which included scale anchors for all response options, when several other scales and scale formats are used by COS developers. Additionally, participants were only asked about ease of use, understanding and clarity of scales at the end of round 3 of the Delphi. This decision was taken to avoid overwhelming participants with a lengthy survey at each round. As a result, we do not have data on these metrics from participants who dropped out in round 1 and round 2. Future work is warranted to explore the user experience across rounds with different scales. Furthermore, the proportion of outcomes rated as “important” in each round and the preliminary COS on both scales depends on the chosen definition of consensus. Therefore, the reproducibility of this study in other fields of COS development, focussing on other health related topics, different scales, larger samples and different consensus definitions is warranted. Finally, as previously mentioned, only one patient participated in our face-to-face consensus meeting due to circumstances out of our control (i.e. flight cancellations). We did, however, have members who identified with multiple groups (i.e. a researcher/patient and clinician/patient) so the patient voice was not unheard. We do not believe this influenced the final COS as all participants had the opportunity to discuss the outcomes and the facilitator actively encouraged comments on each outcome from all attendees. Equally all attendees voted independently at the consensus meeting thus ensuring stakeholder group input into all outcomes that made up the final COS.

## Conclusions

Overall, our results identified that outcomes derived from patient interviews did not directly impact the final COS in this study, but the scoring scale used to prioritise outcomes did, highlighting further methodological considerations and challenges when developing COS protocols. While this study shows that a 5-point scale may be recommended to use in Delphi consensus methods in terms of impact on outcome selection and ease of use and clarity, we acknowledge that this is one study only, and, as such, definitive conclusions cannot be drawn. Future research concerning the impact of patient-derived interviews outcomes on the final COS, overall patient involvement throughout the consensus methods and comparisons of other rating scales on the final outcome selection is needed.

## Supplementary Information

**Additional file 1.** PGP-COS Outcome Voting Each Round.

## Data Availability

Not applicable. This methodological study was embedded in the PGP-COS study; no additional data was generated for this study. However, data for the main PGP-COS study is available by request by contacting the lead author Dr. Alexandria Remus.

## Abbreviations

COS: Core outcome set
PGP-COS: Pelvic girdle pain core outcome set
COMET: Core outcome measures in effectiveness trials
GRADE: Grading of recommendations assessment, development and evaluation
